# “Great balls on fire:” known algorithm with a new instrument?

**DOI:** 10.1080/17453674.2020.1840029

**Published:** 2020-11-04

**Authors:** Stephan M Röhrl

**Affiliations:** Division of Orthopaedic Surgery, Oslo University Hospital, Oslo, Norway

I am sure Jerry Lee Lewis would excuse my witticism with the title of his famous rock ’n roll song. However, it depicts metaphorically that radiostereometric analysis (RSA) has become even more of a hot topic lately.

You might either love or be frustrated about RSA. Journal editors and study investigators love this high-precision measuring method for its high resolution and objectivity, but researchers sometimes despair over the meticulous working steps and processing of data necessary in order not to lose any precious study patients. Implant companies are often positive toward RSA studies but reluctant to mark a batch of implants with beads directly in the production process. Nevertheless, no other imaging entity can so far beat the precision and accuracy of RSA in spite of the great advances of MRI and CT. Since its introduction by Selvik (1989) RSA has over the past 40 years evolved to be the most trusted tool to monitor implant migration just like an orthopedic GPS separating stable and unstable implants or measuring material wear to the tenth of a millimeter. Meanwhile RSA has gained an almost religious status through its ability to predict the survival of implants by extrapolating migration data after a short time in situ (Kärrholm 2012). As far back as the 1990s, Kärrholm showed the predictive value for late failure of early migration in cemented stems for the hip (Kärr­holm et al. 1994). By now the evidence for similar patterns is also increasing for acetabular cups (Pijls et al. 2012) and for selected uncemented hip stems (de Vries et al. 2014) as well as for tibial components in total knee arthroplasty (Pijls et al. 2018). However, in general the interpretation of early migration of uncemented implants seems to be more complicated. We do not know for how long after implantation migration of uncemented hip and knee components can last without compromising long-lasting stability. With regard to other types of artificial joints (e.g., in the upper extremity) the threshold for acceptable early migration is still poorly documented. Nevertheless, it is obvious that continuous migration of an implant will eventually end in painful clinical loosening.

RSA studies can disclose the success or failure of an implant and thereby save thousands of unaware patients from poorly performing implants. This is strongly needed as the revelation of “implant files” (Lenzer et al. 2018) has shown the insufficiency of preclinical implant testing. If the implants are faulty it also puts the surgeon in the line of fire despite his or her treatment with good intentions. Several authors have therefore outlined the importance of RSA in the quest for stepwise and safe introduction of new implants (Malchau 2000, Nelissen et al. 2011).

The International Radiostereometry Society and other pioneers are still working hard to standardize the execution, analysis, and presentation of RSA results to render the method useful for orthopedic surgeons all over the world (Kärrholm et al. 1997, Ryd et al. 2000, Valstar et al. 2005). Just recently Pijls (2020) commented on the positive effect of standardization of RSA, which even resulted in an ISO norm 16087:2013(E). Over 700 studies are registered in PubMed with *Acta Orthopaedica* as the primary platform with over 400 articles alone.

Would it not therefore be handy to perform a reliable migration study for any implant or even follow up each patient with RSA? An appealing thought, but although digital RSA has become a lot more user friendly compared with the times when the data was stored on punch cards or markers were marked manually, it is still an invasive method and has not yet found its way into the clinical workday as a diagnostic tool except in some RSA centers (Horsager et al. 2017). Sophisticated edge-detection software has mostly obliviated the need for markers in implants (Kaptein et al. 2003, Lindgren et al. 2020) but the human bone still has to be marked with tantalum markers as reference during the surgery.

This issue of Acta includes an article from van der Voort et al. (2020) with 25 years’ RSA follow-up, which is the longest of its kind ever. An uncemented stem with 3 different coatings is stable irrespective of implant surface used. The article illustrates the pitfalls and lessons learned with clinical RSA studies as mentioned above. A second article by Sandberg et al. (2020), also in this issue, reports the migration pattern of a new uncemented femoral stem analyzed with a new method based on CT. Likewise, as earlier RSA studies of other stems have shown, after initial minimal migration the stem settles and is stable. Can we rely on this data?

Since the turn of the millennium a group of researchers in Sweden have explored the possibilities of providing similar migration data with CT as with RSA. Olivecrona et al. (2002) showed in the hip that it is in principal possible to perform migration and wear measurements with CT. Initially the researchers still used markers to identify the bone as a reference (Olivecrona et al. 2004, Otten et al. 2017) but bony landmarks and implants can be identified by image segmentation and fusion techniques alone without any markers (Noz et al. 2001). Additionally, earlier downsides of CT have changed. First, CT technology has taken a huge step forward in reducing the irradiation dosage for the patients (Sandgren et al. 2016) and second the amount of metal artefacts caused by implants could be reduced considerably by new measures (Lell et al. 2013, Wellenberg et al. 2016). These developments might have become a game changer for the group around Olivecrona and Weidenhjelm, who have teamed up with a strong imaging company to develop a software interface suitable for radiologists and orthopedic surgeons with a special interest (Olivecrona et al. 2004, Jedenmalm et al. 2011, Maguire et al. 2014, Svedmark et al. 2015, Otten et al. 2017, Eriksson et al. 2019, Broden et al. 2020b).

Low-dose CT provides direct 3-dimensional data, which renders obsolete a cage and double examinations as used in classic RSA. Image segmentation and fusion algorithms even make the use of markers for bone and implant unnecessary. The technology comes in 2 forms: computer tomography motion analysis (CTMA) as migration analysis over time (Broden et al. 2020b) or image motion analysis (IMA) as stability testing on the same day between 2 examinations separated by a provocation (Svedmark et al. 2015). The latter is comparable to 2 RSA examinations on the same day in an unloaded and a loaded position (Bragonzoni et al. 2005, Dunbar et al. 2012, Kibsgard et al. 2017, Lam Tin Cheung et al. 2018). The algorithm and software behind the CT analysis, however, are basically the same.

The potential of this new technology seems vast. CTMA technology might make in vivo testing of new implants available for anyone, which might finally make a stepwise introduction of all new implants feasible.

IMA might open up to become an ubiquitous diagnostic tool for implant loosening or any other type of joint instability, which might give the orthopedic surgeon a more precise indication for surgery. CTMA and IMA have the potential to revolutionize the way we quantify instability. And this not only for the hip but for any other joint—with or without an implant (Olivecrona et al. 2016, Broden et al. 2020a)!

We now have to question whether the time is right for a paradigm shift for in vivo migration measurements. Not quite I would suggest. Before widespread use of this new technology it has to live up to the standards of proven RSA. We have to test and validate this method thoroughly against the existing gold standard RSA because there might be some methodological pitfalls we are not aware of this far.

Metal artifact reduction will not be equally effective for all implants. Type of metal, size, shape, and thickness will probably affect how precisely an implant can be identified (Radzi et al. 2014). Bone is living matter that changes its shape over time. Bony landmarks might therefore change their form and position. Both might lead to loss of precision and accuracy, rendering data useless.

RSA has been an orthopedic domain. Developed by orthopedic surgeons and engineers, it has been performed and driven by orthopedic surgeons with the help of radiographers. CT technology and its data analysis are mainly in the hands of radiologists. This might in the future change the working distribution. Orthopedic surgeons provide the clinical questions and the radiologist provides the measuring method and maybe even the analysis. Also, the industry should have an interest but it might not come away financially as easily as before with ambitious orthopedic surgeons willing to devote years or even their lives to meticulous RSA analysis. All parties will have to find a way to collaborate for the sake of our patients.

Nevertheless, the new CT technique is here and promising. Offering a new tool, it might open up possibilities not yet even imagined. It is now up to the RSA researchers around the world to play with and test this new method down to the bones in every aspect and detail. Maybe we are on the verge of gaining a new useful tool for all orthopedic surgeons?

Referring back to music, no matter what rhythm we play in the future, the algorithm has to be correct. RSA players around the world: let’s test this new instrument!
